# Tree invasions and biosecurity: eco-evolutionary dynamics of hitchhiking fungi

**DOI:** 10.1093/aobpla/plw076

**Published:** 2016-11-07

**Authors:** Treena I. Burgess, Casparus J. Crous, Bernard Slippers, Jarkko Hantula, Michael J. Wingfield

**Affiliations:** 1 Centre of Phytophthora Science and Management, School of Veterinary and Life Science, Murdoch University, Murdoch 6150, Australia; 2 Forestry and Agricultural Biotechnology Institute, University of Pretoria, Pretoria 0002, South Africa; 3 Present address: Centre for Ecology, Evolution and Environmental Changes, Faculty of Sciences, University of Lisbon, Campo Grande, Lisbon 1749-016, Portugal; 4 Natural Resources Institute Finland, Natural Resources and Bioproduction Unit, Vantaa 01300, Finland

**Keywords:** Canker pathogens, eco-evolutionary experience, host shifts, mycorrhiza, novel environments, oomycetes, tree pathogen

## Abstract

When non-native plants reach novel environments, they typically arrive with hidden microbiomes. In general, most of these hitchhikers remain on their co-evolved hosts, some contribute to the invasiveness of their hosts, and a small number can undergo host shifts and move onto native hosts. Invasion success can vary depending upon the different categories of fungal associates. When an invader tree relies on a fungal mutualism to survive in the new environment, there is a fundamentally lower likelihood of either the tree, or the fungus, establishing novel associations. In contrast, parasitic hitchhikers could merely use their host plants to move through the landscape and to become established on new hosts (host shifts). Evidence suggests the frequency of these host shifts is low and depends upon the fungal functional group. However, epidemics caused by invasive pathogens in native ecosystems have occurred globally. Thus, elucidating the potential for hidden non-native fungi to form novel host associations in a new environment is important for biodiversity conservation.

## Abstract (precis)

Non-native plants reach novel environments with hidden fungal hitchhikers. Some remain on their hosts, often contributing to invasiveness while others move beyond and naturalise and some cause epidemics on native trees. Among the three main types of fungal associations, pathotroph, symbiotroph or saprotroph, there are many categories reflecting either the part of the plant with which the fungus forms an association, the type of association, or the type of disease symptom it causes. The likelihood of non-native fungi forming novel host associations in a new environment is dependent upon the type of association.

## Introduction

The negative consequences of plant invasions to ecosystem integrity are well recognized (Richardson and Van Wilgen 2004; Le Maitre *et al.* 2011). However, plants do not arrive alone, and there is an extensive body of literature on the pests and pathogens being co-introduced with non-native flora (Brasier 2008; Wingfield *et al.* 2011; Eschen *et al.* 2015; Ghelardini *et al.* 2016; Burgess and Wingfield 2016). The inadvertent introduction of pests and pathogens is a global phenomenon strongly driven by the growing global trade in live plants (Liebhold *et al.* 2012; Santini *et al.* 2013). Therefore, unsurprisingly, there are loud and on-going calls for action to better regulate plant trade. These seek to ensure both cost-effective biodiversity conservation (Rouget *et al.* 2016) and commodity production (Hantula *et al.* 2014; Wingfield *et al.* 2015).

Largely ignored in the global transportation of plants are the hidden microorganisms, within seed, the plants themselves, or the soil in which the plants were grown. These so-called hitchhikers include those beneficial to their hosts, those detrimental, those that may become naturalized without causing harm, and those negatively impacting native plants via host shift events (Table 1). As discussed in this issue (Zenni *et al.* 2016), the term “second genome”, recognising the impact of associated microbes on the phenotype of organisms, is also applicable to plant invasions, where success is due not only to plant traits, but also those of the associated microorganisms. Thus, hitchhikers, not only have the ability to promote the invasion success of their hosts in novel environments, but they potentially also threaten native ecosystem functioning through host-shifts.

**Table 1:** Terminology used in this review.

Term	Definition
Introduced^1^	A recently introduced species
Naturalized^1^	A self sustaining population of an alien species (evidence for reproduction)
Invasive^1^	A self-sustaining population with evidence of spread or impact
Spread^1^	An alien fungal species that has spread across a landscape
Impact^1^	An introduced fungal species that causes disease on native flora or outcompetes native fungi and threatens biological diversity
Host-shift	An invasive pathogen moving from its non-native host to an native host or visa versa
Hitchhikers	Fungi transported with asymptomatic plants (co-invaders), including pathogens (parasites)

^1^Based on definitions in common use (Blackburn et al. 2011; Richardson et al. 2011; Pereyra 2016).

Applying eco-evolutionary experience (EEE) principles to novel host-microbe interactions (sensu Saul and Jeschke 2015) can help predict the invasion risk of hitchhikers. This concept describes the idea that introduced organisms, not evolved or adapted to the introduced landscape, could still possess the ecological and evolutionary traits allowing them to establish beneficial biotic interactions in the new environment. An example for fungi would be a pathogen of an introduced tree species shifting hosts onto a phylogenetically related native tree species in the new environment. Conversely, if the native pathogens do not have high EEE with the introduced tree species (e.g. competition or predation), the risk of spreading of these non-native organisms (both tree and pathogen) increases considerably (Facon *et al.* 2006; Saul *et al.* 2013; Saul and Jeschke 2015). The interrelationship between the EEE of the tree host and its fungal associates remains unclear. For example, when a tree is introduced, but fails to establish due to low EEE in the novel environment (e.g. low resistance to attack by native enemies), could hitchhikers still establish and spread independently?

Fungi can be classified into three broad trophic levels: (1) pathotrophs which receive nutrition by harming host cells, (2) symbiotrophs which receive nutrition by exchanging resources with host cells and (3) saprotrophs which obtain nutrient by breaking down dead host cells (Tedersoo *et al.* 2014). However, within these broads trophic levels, there are many categories (functional groups or guilds) reflecting either the part of the plant with which the fungus forms an association, the type of association, or the type of disease symptom it causes (Nguyen *et al.* 2016). Thus, the terms symbiont, mutualist or parasite, as used in the invasion biology literature (Richardson *et al.* 2000; Torchin and Mitchell 2004; Mitchell *et al.* 2006; Blackburn and Ewen 2016), do not recognize the nuances of the different associations fungi have with their hosts. Here we consider fungal functional groups with ten different life history strategies, providing examples for these tree associated fungi (including oomycetes), their transport and introduction, and, using the EEE platform, predict the potential of the different groups to establish and spread into the natural environment. In order to interrogate these theories, the focus of this communication is on the global transport of fungi (and oomycetes) with long-lived tree species, because they harbour all these groups of fungal associates. We recognize the importance of other microorganisms like bacteria in the invasion success of trees (Le Roux *et al.* 2016), but this will not be covered here.

## Multiple Fungal Associates: The Hidden Threats of Tree Invasions

### Endophytes

Endophytic fungi exist within host tissues without causing any obvious symptoms (Saikkonen 2007; Rodriguez *et al.* 2009). This group of fungi are most commonly transported along with their hosts (Aschehoug *et al.* 2012), however, relatively little is known regarding the diversity and biology of endophytic fungi, as many are unculturable (Sun and Guo 2012). Although some endophytes have been identified, their roles (in general, and in invasion biology), specificity and ability to move between host species are poorly understood. There are numerous recent studies using high throughput sequencing technologies considering the diversity of endophyte communities in woody plants (e.g. Kemler *et al.* 2013). However, to date, these studies while highlighting the extraordinary diversity of micro-organisms harboured within a plant cannot provide insights into the function of these endophytes.

The group of endophytes for which the most is known are those Class 3 endophytes (Rodriguez *et al.* 2009) classified as latent pathogens (Carroll 1988), such as members of the Botryosphaeriaceae (Slippers and Wingfield 2007; Phillips *et al.* 2013). Normally, these fungi are benign, but when host plants are stressed, they can cause serious disease problems (Slippers and Wingfield 2007). Where invading trees are planted at the limits of their climatic range (and thus stressed), or as the climate changes, the negative impact of endophytic latent pathogens on tree survival will become important. Tree endophytes in the Botryosphaeriaceae are not typically transmitted by seed, but are rather acquired horizontally from the environment as seedlings emerge (Burgess and Wingfield 2002; Bihon *et al.* 2011a). Seeds developing in nurseries will thus be free from these common endophytes while those found beneath adult trees acquire them soon after germination (Ganley and Newcombe 2006; Bihon *et al.* 2011b). Many species in this group have wide host ranges and are known to move between host species, from exotics to natives and *visa versa*. For example, in Uruguay, the known Australian eucalypt endophyte*, Neofusicoccum eucalyptorum*, has been isolated from both introduced eucalypts and native Myrtales (Pérez *et al.* 2010). Similarly, *Neofusicoccum parvum*, a common endophyte of many woody plants is associated with trees in horticulture, trees in plantations and native forest trees on many continents (Sakalidis *et al.* 2013). The endophytic communities of Siberian larch (*Larix sibirica*) were most diverse in locations where the tree species had been introduced centuries ago and not in the area of its natural distribution, as it had acquired many associates from the new environment (Kauhanen *et al.* 2006). Endophytes represent a functional group of plant-associated hitchhikers, which are known to both move onto native vegetation, however, introduced plants also acquire endophytes from the new environment.

### Arbuscular mycorrhizal fungi

Most land plants form mutually beneficial mycorrhizal associations with fungi, and many of these associations are obligate. Arbuscular mycorrhizal fungi (AMF) have broad hosts’ ranges (Öpik *et al.* 2010) and global distributions. Biogeography, climate and plant community composition can affect the diversity of AMF communities, but account for only a small amount of the observed variance (Kivlin *et al.* 2011; Öpik *et al.* 2013). There is a considerable body of literature considering the role of AMF in facilitating invasion of exotic plant species (Shah *et al.* 2009). However, it is unclear whether the AMF have been introduced with the invasive plant species, or acquired in the new environment. Additionally, the success of the invasive plants may be in part due to positive plant-soil biota feedback in the new environment (Reinhart and Callaway 2006). In fact, it is considered highly likely that introduced plants would find and acquire new AMF partners (Richardson *et al.* 2000).

### Ectomycorrhizal fungi

Many ectomycorrhizal fungi (EMF) display a degree of specificity (Tedersoo *et al.* 2014), and alien trees often have lower species diversity and functional diversity of hyphal foraging strategies than those of native trees (Dickie et. al). In forestry, it has long been recognized that the establishment of pine plantations in the southern hemisphere (SH) required an association with an appropriate EMF (Kessell 1927; Rayner 1934) as none of the numerous local southern hemisphere EMF were able to provide appropriate associations. Thus, when establishing new nurseries, seedlings were often inoculated with soil from established nurseries or healthy plantations (Kessell 1927). Consequently, almost all EMF found associated with SH pine plantations have a natural distribution in Europe or North America (Dunstan *et al.* 1988). EMF not adapted to eucalypts fail to form effective mycorrhiza (Burgess *et al.* 1994), and thus the establishment of eucalypt plantations in Asia was facilitated through the introduction of ectomycorrhizal fungi from their native range in Australia (Brundrett *et al.* 1996). In Spain, Australian EMF promote eucalypt invasiveness (Díez 2005). Mycorrhizal fungi, as root inhabitants, can only be transported with their hosts where the plants are rooted in soil or when the fungi have been deliberately introduced. Thus, host-specific EMF are often required for successful invasions by their hosts in new environments (Hayward *et al.* 2015). However, there are only a few documented cases of introduced species of EMF moving onto native hosts, and the effects on native EMF remain unknown (Nuñez and Dickie 2014).

### Leaf and shoot pathogens

Many generalist necrotrophic pathogens are opportunistic, entering leaves through wounds or living on exudates found on leaf surfaces, and these can be acquired in the new environment. However, there are also numerous host-specific leaf pathogens, or at least infect only related plant species (Parker *et al.* 2015). For example, the best-known pathogens of plantation forestry trees in the SH belong to the Teratosphaeriaceae and Mycosphaerellaceae (Hunter *et al.* 2011). They have been moved globally with their hosts or caught up after initial establishment (Crous *et al.* 2016), and can often cause severe disease problems in plantations. Dothistroma needle blight of pines can be a devastating disease in SH plantations following introduction, but there is no evidence of host shifts onto native gymnosperms (Barnes *et al.* 2004). Similarly, one of the most devastating foliar pathogens of eucalypts, *Teratosphaeria nubilosa*, has been distributed globally with its hosts, but has never moved onto native vegetation (Pérez *et al.* 2012). Many leaf pathogens have been co-introduced with their hosts, and they must, in most cases, have been transported on infected plant material (Burgess and Wingfield 2016). The host specificity of many of these pathogens implies it might be possible to use them as biological control agents for invasive plant species.

### Rust pathogens

Rust fungi are usually highly host specific; however, there are also examples where the rusts themselves have undergone host shifts very soon after introduction (Wingfield *et al.* 2015). A vivid example of a tree rust pathogen moving globally on plant products is the autoecious *Puccinia psidii*, the causal agent of the disease known as myrtle rust. This pathogen of native Myrtaceae in South America (Glen *et al.* 2007) was introduced to Australia in 2010 and quickly became invasive as it moved along the eastern seaboard where many native Myrtaceae have no resistance (Carnegie *et al.* 2016). White pine blister rust, *Cronartium ribicola*, a heteroecious species, is native to northern Asia where the local white pines are resistant, but rapidly became invasive on native white pines following its introduction into north America (Maloy 1997). In both these examples, the pathogen host-shifts were limited to phylogenetically related hosts. For white pine blister rust, this was limited to a few species of five needle pines, while for myrtle rust, host shifts were much broader, but restricted to the family Myrtaceae.

When a rust fungus is highly host specific, they are valued biological control agents of invasive plant species. For example, *Uromycladium tepperianum*, a host-specific rust fungus of *Acacia saligna*, was introduced and successfully deployed to control the invasion of this tree in fynbos landscapes in South Africa (Wood and Morris 2007). It should be noted that a pathogen used as a biological control agent is itself an invasive organism.

### Canker pathogens

Canker pathogens produce lesions on the stems of trees where they typically enter via wounds, subsequently infecting the cambial tissues. They include genera and species of fungi exhibiting varying degrees of specificity ranging from highly host specific to those with relatively wide host ranges (Sinclair and Lyon 2005). Because these fungi infect and express symptoms in older plants, it might be expected they would rarely be transported. However, canker pathogens make up the largest group of invasive forest pathogens in Europe (Santini *et al.* 2013). The best-known invasive tree pathogen is the devastating canker pathogen *Cryphonectria parasitica*, the causal agent of chestnut blight in Europe and North America (Anagnostakis 1987). The pathogen was introduced from Asia, probably Japan, where local chestnut species are naturally resistant (Milgroom *et al.* 1996). *Fusarium circinatum* causes pitch canker of *Pinus radiata* both in its native range in California and in some regions where *P. radiata* is planted as an exotic. This pathogen is thought to originate from related *Pinus* spp. in Mexico (Gordon 2006). Conversely, while there are numerous serious canker pathogens of exotic eucalypts such as *Chrysoporthe cubensis* and *Teratosphaeria zuluensis*, none of these have been shown to exist in the native range of the genus indicating that these pathogens have been acquired in the new environment via host-shifts (Burgess and Wingfield 2016).

### Wilt pathogens

Wilt pathogens enter the vascular systems of their hosts and thus disrupt normal water flow leading to wilting. Most wilt pathogens, especially those of trees, require wounds for infection and many are associated with insect vectors producing sites of ingress (Wingfield *et al.* 2016). Wilt pathogens cause some of the best-documented invasive diseases including the infamous Dutch Elm Disease caused by *Ophiostoma ulmi* and *O. novo-ulmi* in Europe and North America (Brasier 1991; Hubbes 1999). Thought to be native to North America where *Platanus occidentalis* is resistant, the introduction of *Ceratocystis platani* to Europe has been devastating for the native *P. orientalis* (Ocasio-Morales *et al.* 2007). *Ceratocystis fimbriata s.l.* a common wilt pathogen of many crops and plantation tree species, has rarely impacted in natural ecosystems, however, post introduction and naturalization in Hawaii, it has now become a major pathogen of the native tree species *Metrosideros polymorpha* (Mortenson *et al.* 2016). The most common pathways for introduction of these pathogens has been with their insect vectors, many of which can be found in widely traded wood and wood products (Haack 2006; Roques *et al.* 2010), or with contaminated soil (Liebhold *et al.* 2012).

### Rot pathogens

Rot pathogens are those capable of infecting and rotting the wood of healthy trees. Because symptoms are quite obvious, and they generally only develop on older trees, they are not obvious hitchhikers. Common genera such as *Phellinus* and *Ganoderma* are distributed globally. There have been limited studies on their phylogeography; however, isolation-by-distance for southern hemisphere *Ganoderma* spp. has been demonstrated (Moncalvo and Buchanan 2008), indicating that many rot pathogens of invasive trees have not been transported, but rather acquired from the new environment.

Prior to the advent of molecular systematics, the well-known rot (and soil) pathogen, the honey fungus *Armillaria mellea*, was considered to have a global distribution. It is now known that there are many species of *Armillaria* having separate continental distributions (Coetzee *et al.* 2000). However, there is an intriguing example of the introduced *A. mellea* establishing and spreading into parks and gardens of the South African Cape Peninsula; this species was likely introduced in the 1600s and it remains a problem today (Coetzee *et al.* 2001). This introduction most likely occurred via the transport of potted trees, where the pathogen would have been resident in rotten root debris.

The tree root pathogen *Heterobasidium annosum* was original thought to have a broad distribution across the northern hemisphere until, as with *Armillaria mellea*, molecular systematics and detailed mating studies revealed there were several species with distinct continental distributions (Garbelotto and Gonthier 2013). *Heterobasidum annosum* has a natural distribution in North America, but was introduced into Italy (during the second world war, probably on wood packaging), where it caused more damage on the native *Pinus pinea* (stone pine) than the closely related local pathogenic species, *H. annosum*. The continuous spore release by the invasive species compared with seasonal release by the native *H. annosum* contributes to its success (Garbelotto *et al.* 2010). The North American species is also hybridizing with the local species resulting in novel genetic combinations with unpredictable phenotypes (D´Amico Motta *et al.* 2007; Gonthier and Garbelotto 2011). Thus, while introductions of rot pathogens occur relatively rarely, where they have been introduced with wood packaging or infected soil, they can naturalize and become invasive.

### Saprotrophs

Saprotrophic fungi derive their nutrients from dead plant material. They may have been transported globally with their hosts, and because they have limited nutritional needs and do not need to overcome the defence strategies of living plants, they should readily naturalize. This group of fungi would typically be considered as neutral hitchhikers. One example is *Clathrus archeri*, a saprotrophic fungus native to Australia, now found globally, with its movement most probably via contaminated soil. Not surprisingly, as a saprotroph, there have been no obvious negative effects to tree health, with the only signs of presence due to the unpleasant odour it produces (Desprez-Loustau 2009). Similarly, the saprophyte *Favolaschia calocera*, first described from Madagascar, has since been reported in several countries including Italy, Australia and New Zealand (Vizzini *et al.* 2009). While the species has become wide spread, it is more common on disturbed sites, and is considered a poor competitor against native wood-inhabiting fungi. Introduced and naturalized saprophytic fungi will mostly remain undetected unless they obviously outcompete a native saprophytic fungal species.

### Soil pathogens (oomycetes)

Pathogens in this category are moved globally to new environments in contaminated soils. Intentional movement of soil with potted plants was evidently common in the past, for instance during early colonization and the transport of fruit and other trees. Although this is incomprehensible in terms of plant quarantine, large amenity plants continue to be produced in one region and transported to another along with large volumes of soil (Brasier 2008). Soil pathogens often go undetected because they fail to produce obvious symptoms during the early stages of infection. The most commonly moved tree pathogens globally today are species of *Phytophthora* (Brasier 2008). They are often detected in asymptomatic nursery plants (Migliorini *et al.* 2015). Moreover, they may even be transported as hitchhikers on non-hosts. For example, in Australia, *Phytophthora cinnamomi* is the most devastating introduced pathogen and a key threat to the biodiversity of the megadiverse vegetation in the south-west botanical hotspot (Davison and Shearer 1989). This pathogen was accidentally introduced into Australia on tolerant or asymptomatic hosts used to establish orchards (Zentmyer 1985) and its global distribution has probably followed the same pattern (Burgess *et al.* 2016).

## Fungal Associates and Tree Invasion Success

The introduction of mutualists, such as mycorrhizal fungi, can contribute to the rapid establishment and spread of the non-native tree species (Dickie *et al.* 2010; Zenni *et al.* 2016) (Table 2). Here, the high EEE of the tree-host in the novel environment is directly influenced by the presence of the fungal mutualist. However, if the fungus and the host are introduced separately, especially into landscapes where the resident flora is phylogenetically distinct (Parker and Gilbert 2004), then each may have low EEE. Based on the evolutionary dynamics necessary to form close mutualisms, co-introduced fungal mutalists should have a fundamentally lower likelihood of spreading onto native hosts. However, a low likelihood of establishment and spread might only be true for host-specific mutualistic fungi, since more general or opportunistic mutualistic fungi could have higher EEE when introduced into regions where the resident flora is phylogenetically similar. Endophytes, the group of fungi most likely to arrive as hitchhikers, vary in their influence on tree invasions (Table 2). In fact, while very little is known about the benefits of endophytes in general, the sub-group known to be latent pathogens (Rodriguez *et al.* 2009) will cause disease in their hosts if they are introduced into unsuitable environments.

**Table 2:** Documented impacts of fungal hitchhikers on the success of tree invasions and on the invaded ecosystem.

	Success of tree invasions		Impact on invaded ecosystem	
	Positive^1^	Negative^2^	Negative^3^	Neutral^4^
Arbuscular mycorrhiza	**+**			**+**
Ectomycorrhiza	**+**			**+**
Endophytes	**+**	**+**		**+**
Leaf pathogens		**+**		**+**
Canker pathogens		**+**	**+**	**+**
Wilt pathogens		**+**	**+**	**+**
Rust pathogens		**+**	**+**	**+**
Rot pathogens			**+**	**+**
Oomycetes		**+**	**+**	**+**
Saprophytes				**+**

^1^Facilitate host invasion.

^2^Cause disease on introduced host and could limit host invasion.

^3^Undergo host shifts and cause disease in new environment.

^4^Naturalize and integrate into existing networks without obvious impact.

The success of tree invasions has often been linked to escape from its natural enemies (Keane and Crawley 2002), including plant pathogens, all of which would impact negatively on potential invasion success (Table 2). However, pathogens usually catch-up to their hosts over time (Crous *et al.* 2016). Additionally, native pathogens can eventually move onto the introduced trees. This is especially evident if components of the resident flora are closely related to the introduced tree species. For example, in Europe, phylogenetically related naturalized and native tree species shared similar numbers of interactions with similar types of fungal pathogens (Vacher *et al.* 2010). Thus, several centuries after introduction, the network has assimilated the naturalized species, both the trees and their pathogens. It could be argued that phylogenetically distinct tree species would have a greater chance of becoming invasive as they would attract fewer native pathogens.

## Host-Shifts in New Environments

Introduced fungi, especially those remaining associated with their naturalized or invasive hosts, often have a neutral effect on native plant communities (Table 2). However, there are documented examples of numerous introduced plant pathogens undergoing host shifts onto, and impacting on native tree species. Among these pathogens, oomycetes and those classified as canker or wilt pathogens have had the greatest impacts (Table 2). Invasive pathogens resulting in novel disease associations are generally those transported between regions naturally harbouring related hosts in similar ecosystems; i.e. across the northern hemisphere (Hansen 2008; Loo 2009; Burgess and Wingfield 2016; Ghelardini *et al.* 2016; Müller *et al.* 2016). These fungi have been co-introduced with hosts from a different continent and have moved onto related species (naïve hosts) having little resistance (Müller *et al.* 2016). They often do not adversely affect the health of their co-evolved hosts and have thus used their hosts’ EEE to establish as a pathway to invasion. In contrast, disease outbreaks in the SH tend to be on exotic tree species, mostly those introduced for forestry (Wingfield *et al.* 2015; Burgess and Wingfield 2016). Disease outbreaks of invasive pathogens in native ecosystems in the SH are rare, with the most extensive and best recorded being the invasive oomycete, *P. cinnamomi*. Oomycetes most often arrive as hitchhikers with infected plant material or with contaminated soil. Therefore, early detection and the rapid eradication of invasive plants populations is critical because their persistence in the landscape may allow the time for hidden fungi to increase their fitness on related native species.

## Conclusions

While numerous fungi have been introduced along with non-native trees globally, most appear to remain on their co-evolved hosts; some contribute to the invasiveness of their hosts and only a few undergo host-shifts onto native plants. Nonetheless, the hitchhikers of tree invasions, the hidden non-native fungi, represent a neglected research field, and this is in need of critical redress. It is easy to see how these fungi not only promote the success of invasive trees in the novel environments, but due to host-shift potential, could increase the future invasion debt in recipient countries (Rouget *et al.* 2016).

The necessary eco-evolutionary characteristics to facilitate invasion success can vary between different groups of fungal associates and their hosts. Predicting the likelihood of hitchhikers forming novel interactions in the invaded range must, therefore, depend strongly on the phylogenetic relatedness and EEE of introduced genera to the flora in the recipient community (Parker and Gilbert 2004; Saul *et al.* 2013; Saul and Jeschke 2015). The fact that EEE principles can help explain both tree and fungal associate interactions in the novel and related landscapes, suggests much more attention should have been taken historically to minimize the introduction of related plants into recipient ecosystems. With this knowledge, the global trade in plants can, and should, be better regulated.

## Sources of Funding

This paper had its origin at a workshop on “Evolutionary dynamics of tree invasions’’ hosted by the DST-NRF Centre of Excellence for Invasion Biology (C•I•B) in Stellenbosch, South Africa, in November 2015. Funding for the workshop was provided by the C•I•B, Stellenbosch University (through the office of the Vice Rector: Research, Innovation and Postgraduate Studies), and the South African National Research Foundation (DVGR Grant no. 98182). Murdoch University through the Sir Walter Murdoch Scheme (awarded to M.W.) supported the travel of T.B. to attend the workshop.

## Contributions by the Authors

All authors contributed to discussions and subsequent writing of the manuscript.

## Conflict of Interest Statement

None declared.
